# The Effect of Tree Width on Thoracolumbar and Limb Kinematics, Saddle Pressure Distribution, and Thoracolumbar Dimensions in Sports Horses in Trot and Canter

**DOI:** 10.3390/ani9100842

**Published:** 2019-10-21

**Authors:** Russell MacKechnie-Guire, Erik MacKechnie-Guire, Vanessa Fairfax, Diana Fisher, Mark Fisher, Thilo Pfau

**Affiliations:** 1 Centaur Biomechanics, 25 Oaktree Close, Moreton Morrell, Warwickshire CV35 9BB, UK; Erikmac@aol.com; 2 Department of Clinical Science and Services, The Royal Veterinary College, Hawkshead Lane, North Mymms, Hatfield AL9 7TA, UK; tpfau@rvc.ac.uk; 3 FairfaxSaddles, The Saddlery, Fryers Road, Bloxwich, Walsall, West Midlands WS3 2XJ, UK; vanessa.fairfax@fairfaxsaddles.com; 4 Woolcroft Saddlery, Mays Lane, Wisbech PE13 5BU, UK; dianafisher007@yahoo.co.uk (D.F.); woolcroft2002@yahoo.co.uk (M.F.)

**Keywords:** thoracolumbar region, saddle fitting, locomotion, equine

## Abstract

**Simple Summary:**

Determining the correct saddle fit is essential in order to optimise the interaction between the horse and rider dyad, and to reduce the risk of back-related problems or loss of performance as a result of incorrect saddle fit. Although there are industry guidelines (Society of Master Saddlers) on correct saddle fit, some saddle fitters (and others) choose to fit saddles that are wider than industry guidelines on the assumption that increased saddle width will enhance equine locomotion and allow the horses’ thoracolumbar spine to function unhindered. This study quantified the effect that a saddle that was one width fitting wider and narrower (based on the Society of Master Saddlers industry guidelines) had on the kinematics of the thoracolumbar spine, thoracolumbar epaxial musculature profiles, equine locomotion, and saddle pressure distribution. It was found that a saddle that was one width fitting wider and narrower affected the kinematics of the thoracolumbar spine, resulting in concavities in epaxial musculature at T13 when using the wide saddle and at T18 when using the narrow saddle. The wide saddle caused areas of high pressures in the cranial region of the saddle and the narrow saddle caused areas of high pressures in the caudal region of the saddle. It is essential that the correct saddle fit is achieved for each horse and rider combination in order to optimise the horse-rider system and reduce the risk of back-related problems or loss of performance that may occur as a result of incorrect saddle fit.

**Abstract:**

This study evaluated the effect of saddle tree width on thoracolumbar and limb kinematics, saddle pressure distribution, and thoracolumbar epaxial musculature dimensions. Correctly fitted saddles were fitted by a Society of Master Saddler Qualified Saddle Fitter in fourteen sports horses (mean ± SD age 12 ± 8.77 years, height 1.65 ± 0.94 m), and were altered to one width fitting wider and narrower. Horses were equipped with skin markers, inertial measurement units, and a pressure mat beneath the saddle. Differences in saddle pressure distribution, as well as limb and thoracolumbosacral kinematics between saddle widths were investigated using a general linear model with Bonferroni adjusted alpha (*p* ≤ 0.05). Compared with the correct saddle width, in trot, in the wide saddle, an 8.5% increase in peak pressures was found in the cranial region of the saddle (*p* = 0.003), a 14% reduction in thoracolumbar dimensions at T13 (*p* = 0.02), and a 6% decrease in the T13 range of motion in the mediolateral direction (*p* = 0.02). In the narrow saddle, a 14% increase in peak pressures was found in the caudal region of the saddle (*p* = 0.01), an 8% decrease in the range of motion of T13 in the mediolateral direction (*p* = 0.004), and a 6% decrease in the vertical direction (*p* = 0.004) of T13. Compared with the correct saddle width, in canter, in the wide saddle, axial rotation decreased by 1% at T5 (*p* = 0.03) with an 5% increase at T13 (*p* = 0.04) and a 5% increase at L3 (*p* = 0.03). Peak pressures increased by 4% (*p* = 0.002) in the cranial region of the wide saddle. Altering the saddle fit had an effect on thoracolumbar kinematics and saddle pressure distribution; hence, correct saddle fit is essential to provide unhindered locomotion.

## 1. Introduction

Correct saddle fit should enhance the athletic performance [1] of both the horse and rider. Incorrect saddle fit is thought to be a contributory factor towards equine thoracolumbar dysfunction, poor attitude to work, and poor performance [1,2,3,4,5,6,7] in the horse. During locomotion, the equine spine undergoes three-dimensional translational (dorsoventral, mediolateral, and craniocaudal) and three-dimensional rotational (axial rotation, lateral bending, and flexion-extension) movement [8,9,10,11,12]. Essentially, the thoracolumbar region is a dynamic platform on which a saddle needs to be positioned without causing hindrance or restriction to the horse. The underside of the saddle should conform to the dynamics of the horse’s thoracic region and the upper side should conform to the rider’s pelvis and thighs [13].

Generally, saddle fitting is subjective and relies on the skills of an individual to assess the suitability of a saddle for both the horse and rider. In an attempt to standardise saddle fitting, the Society of Master Saddlers (SMS) has produced guidelines that qualified saddle fitters follow. Initially, a static saddle fitting assessment is performed featuring several criteria, as described elsewhere [14]. Once each criterion is met, a dynamic ridden assessment is performed, requiring the saddle fitter to observe the horse and rider in all gaits and in both directions. Tree width is one of the criteria used to determine the correct saddle width (here on, width). Industry guidelines (Society of Master Saddlers) state that the angle of the tree points should correspond to the angle of the horse’s back 5 cm from the caudal edge of the scapula in the static horse. Anecdotally, some saddle fitters (and others), despite industry guidelines, prefer to fit a saddle wider on the assumption that this will allow increased movement of the scapula and of the thoracic spine, both of which are not supported by any scientific evidence. Previously, the agreement between a cohort of Society of Master Saddlers Qualified Saddle Fitters (SMSQSF) when performing a static saddle fitting assessment was reported. Moderate to substantial agreement between saddle fitters was found for eight of the static saddle fitting criteria, that is, (8/10), while only slight (51%, k 0.12) agreement was found for tree width and tree length [14].

Under laboratory conditions, compared with a saddle that was of the correct tree width (defined as a saddle with the lowest overall force in walk and trot), saddles that were one width fitting narrower, one width fitting wider, and two width fittings wider have been reported [3]. Compared with the saddle with the lowest overall force, pressure distribution was found to be higher in the caudal third of the saddle when using a narrow saddle. In the wide and very wide saddles, areas of high pressure were reported for the middle transversal third of the saddle, close to the midline of the equine spine, below where the rider’s centre of mass is positioned at T10–T13 [3]. The region of T10–T13 corresponds to the twist or waist of the saddle. In Grand Prix dressage horses, while trotting, saddles that have a narrow twist or waist have been reported to increase saddle pressures in the region of T10–T13 [15]. These areas of high pressure were associated with reduced carpal and tarsal flexion while trotting [15]. It is speculated that a saddle that is too wide or narrow may result in areas of high pressures, which may have an effect on equine locomotion, limb loading, and epaxial musculature health.

The pitch of a correctly fitted saddle should remain unchanged with a rider, and during locomotion. In a wide saddle, the cranial aspect of the saddle pitches ventrally and induces areas of high pressure beneath the cranial region of the saddle [3]. In a narrow saddle, the caudal aspect of the saddle pitches ventrally, giving the saddle an uphill appearance. Both saddle positions appear to result in high pressures [3], which may lead to unilateral or bilateral concavities in the epaxial musculature in the cranial thoracic region caudal to the scapula [7,16], which horse owners should be aware of as it is speculated that the development of concavities could be used as an indicator of incorrect saddle fit. The pitch of the saddle (wide—cranial aspect of the saddle ventrally positioned; narrow—caudal aspect of the saddle ventrally positioned) will affect the rider’s pelvis and is likely to compromise the effectiveness of the rider seat aids [6].

The aim of this experiment was to quantify the effect of a wide saddle (one width fitting wider than required) and a narrow saddle (fitted one width fitting narrower) on limb kinematics, thoracolumbar kinematics, epaxial musculature dimensions, and saddle pressure distribution, when compared with a saddle fitted following industry guidelines (reported from here on, correct saddle) [17] in horses in trot and canter.

It was hypothesised that, in trot and canter during ridden exercise, a wide saddle would result in the following: (1) an increase in range of motion in the mediolateral direction of the thoracolumbar area; (2) areas of high pressures beneath the cranial portion of the saddle; (3) an increase in front and hind maximum fetlock hyperextension; (4) reduced carpal and tarsal flexion; and (5) concavities in the thoracolumbar musculature in the region of T13. It was hypothesised that the narrow saddle will result in the following: (1) areas of high pressure in the caudal region of the saddle; (2) increased axial rotation of the thoracolumbar region; and (3) concavities in the thoracolumbar musculature in the region of T18.

## 2. Materials and Methods

The study was approved by the Royal Veterinary College ethics and welfare committee, project number URN 20181785-2.

### 2.1. Horses

Fourteen adult non-lame sports horses were used in this study. All horses were warmbloods and of a similar type and conformation, all competing at affiliated dressage (elementary-advanced medium) and showjumping (British Novice—Foxhunter). All horses displayed good muscle definition with a well-defined musculature of the thoracolumbar region. Horses were housed at the same facility. All horses were geldings. They ranged in height at the withers (mean ± SD: of 1.67 ± 0.07 m), body mass (mean ± SD: 553 ± 24 kg), and age (mean ± SD: 9 ± 1 years). Horses underwent a subjective veterinary assessment performed by two veterinary surgeons, including flexion tests of all four limbs, and no lameness was observed. In addition, the horses’ gait asymmetry was quantified using a validated sensor system (Xsens, Enschede, The Netherlands) [18]. Two experienced female riders were used (mean ± SD: height 1.57 ± 0.05 m, body mass mean ± SD: 70 ± 1 kg). Riders were randomly assigned a horse to ride, both riders were right handed and at the time of the study were free from any injuries. Both riders regularly competed in equestrian disciplines, dressage, showjumping, and eventing. In the week preceding the study, both riders had ridden each horse five times for 45 min. Informed consent was obtained from all owners and riders. Riders could withdraw from the study at any point.

### 2.2. Saddles

To limit the effect that a dressage or showjumping saddle design had on the rider, ten new general-purpose saddles (Kent & Masters), with an interchangeable gullet system, were used. For all conditions, a standard high withered saddle cloth (High: 58 cm withers to base, 54 cm lowest point to base of cloth × Wide: 63 cm) was positioned beneath the saddle. Each saddle was fitted by a SMS qualified saddle fitter and ridden in for ten hours prior to the study to allow the flocking (Jacob wool) to settle. Flocking and panel construction were evaluated between each saddle and horse by an SMS qualified saddle fitter. If the flocking had become irregular or compressed, then it was adjusted; these adjustments were performed by a single SMS qualified saddle fitter. The seat size remained the same (17.5″) for all saddles and the horse’s own girth was used throughout, which was a standard non-anatomically shaped girth (Atherstone) with elastic on one side. The girth was fastened, ensuring that the elastic was on the right side. The same stirrup leathers and stirrups were used and the stirrup length remained the same throughout. The order of saddle fit was randomized and blinded to all technicians, riders, and veterinarians (Table 1). At some stage throughout the experiment, each horse was ridden in a correct, wide, and narrow saddle once. A 10° difference between tree widths was used, based on the British Standard for Saddle Trees BS6635/2015, narrow 75° to 84.9°, medium 85° to 94.9°, wide 95° to 104.9°, and ex-wide 105°. Horses were tacked up by the same SMS qualified saddle fitter.

### 2.3. Study Protocol

**Figure 1:**
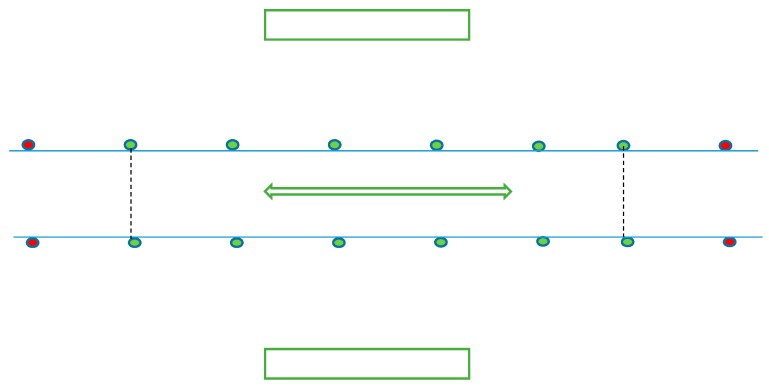
Diagram illustrating the experimental track. The calibrated track is represented by the green markers and the start and end points are represented by the red markers. The calibrated track allowed for eleven consecutive straight motion cycles per repeat.

Each horse underwent a 15 min warm up protocol self-prescribed by the rider for each condition, which included walk, trot, and canter on both the left and right rein. This was followed by a prescribed trot and canter protocol, during which horse kinematics were quantified along with saddle-horse kinetics. Each saddle was evaluated independently by five SMSQSFs following the SMS static and dynamic evaluation guidelines. Data were collected during straight line locomotion in sitting trot and canter on both the left and right rein. All measurements were performed on the same indoor (60 × 20 m) arena on the same surface. The arena dimensions allowed for eleven repeated straight strides, hence eleven were included in the kinematic and kinetic analysis, with both the start and end points being determined using two cones (Figure 1). The surface was levelled prior to, and in between, each trial. Three repeats on the left and right rein were collected within each condition (correct, wide, and narrow). The trial was repeated if the horse lost straightness, tripped, or made an obvious alteration in gait pattern (e.g., shying). Speed was monitored by the same technician using a stopwatch, with start and end points being defined by two markers that were positioned at the start/end of the calibrated track (Figure 1).

### 2.4. Kinematics—Inertial Measurement Units

**Figure 2:**
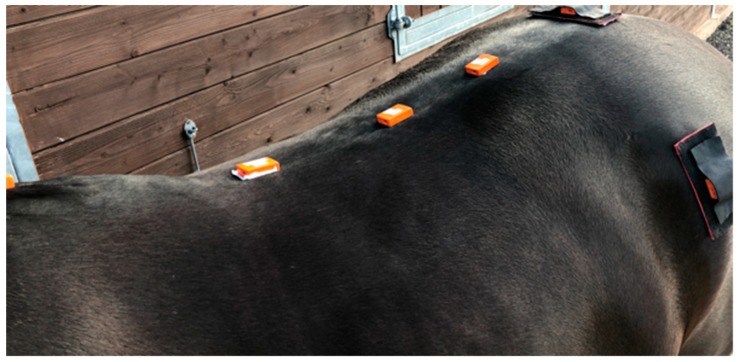
Inertial measurement unit (IMU) sensor locations along the thoracolumbar region. Sensors were glued on to the skin at T5, T13, T18, and L3. Sensor pouches were used for the tubera sacrale (TS) and left and right tuber coxae.

Horses were instrumented with eight MTw inertial measurement units (IMUs) (Xsens) using a validated sensor based system (Xsens MTw,) [18,19]. By manual palpation, sensors were attached over the poll, withers (T5), over the thirteenth (T13) and eighteenth (T18) thoracic vertebra and over the third lumbar (L3) vertebra, between the tubera sacrale (TS), and over the left and right tuber coxae, using custom built pouches and double-sided tape. Sensors along the thoracolumbar spine beneath the saddle (and saddle cloth) representing the T5, T13, T18, and L3 were glued on to the skin using hair extension glue (Figure 2). The same technician applied each sensor throughout the study. Sensor locations were referenced with white skin paint. To reduce variability, sensor pouches remained on the horse throughout each data collection for each saddle width. Sensor data were collected at 60 Hz per individual sensor channel and transmitted via proprietary wireless data transmission protocol (Xsens) to a receiver station (Awinda, Xsens) connected to a laptop computer running MTManager (Xsens) software. IMU specifications: internal sampling rate 1000 Hz; buffer time 30 s; dimensions 47 × 30 × 13 mm; mass 16 g; operating temperature range 0 °C–50 °C; and dynamic accuracy 0.75 degrees root mean square (RMS) (roll/pitch) and 1.5 degrees RMS (heading).

IMU data were processed following published protocols [18]. In brief, tri-axial sensor acceleration data were rotated into a gravity (z: vertical) and horse-based (x: craniocaudal and y: mediolateral) reference frame and double integrated to displacement. Displacement data were segmented into individual strides based on the vertical velocity of the sacrum sensor [20], and median values for the following kinematic variables were calculated over all strides for each exercise condition for all saddle width conditions.

- range of motion: maximum–minimum value over a stride cycle for x, y, and z displacement for trot and canter
- hip hike difference (HHD): difference between vertical upward movement amplitude of left and right tuber coxae during contra-lateral stance [21].

Angular movement (a change in orientation) of T5, T18, and L3 was assessed in three planes; all data outcomes were measured in degrees.

- Flexion-extension range of motion (ROM)—the body’s rotation about the transverse (lateral–lateral) axis.
- Axial rotation ROM—the body’s rotation about the longitudinal (craniocaudal) axis.
- Lateral bending—the body’s rotation about the vertical (dorsoventral) axis.
- Translational movement at T5, T18, and L3 was measured in millimetres in two directions:
  - Vertical direction—up and down movement of the whole horse.
  - Lateral–lateral direction—side to the side movement of the whole horse.

In order to allow interpretation of the effect of tree width, IMU derived kinematic variables were summarized between reins. Range of motion variables were subtracted from each other (left rein value − right rein value) and movement symmetry values (MinD, MaxD, HHD) were added up (left rein value + right rein value). This procedure ensured that for horses performing symmetrically between reins, values near zero are expected, as head and pelvic movement symmetry values show directional circle dependent tendencies (positive for one rein, negative for the other) [22] and ranges of motion would be expected to be near identical for the left and right rein.

### 2.5. Kinetic Data—Pressure Distribution

Kinetic data under the saddle were recorded using a pressure mapping system (MSA600, sampling rate 50 Hz, Pliance System, Novel, Pliance, Ismaninger Str. 51, 81675 München, Germany). The pressure mat consisted of 256 sensors arranged into 8 columns (longitudinal) and 16 rows (transverse), left and right sides of the pressure mat (transverse cells 1–8 representing the cranial region and cells 9–16 representing the caudal region). The mat was divided into two halves with no sensors over the midline of the back. Prior to the study, the pressure mat was calibrated, and during testing (for each horse), the pad was zeroed without the saddle, girth, or rider [23]. It was fitted so that the pressure mat was on top of the horse’s skin and beneath the saddle cloth and saddle, as previously described [15,24,25,26,27]. Peak pressures (kPa) and maximum force (N) (sum of all sensors x area) in trot and canter for all saddle conditions were collected. Our experimental track was positioned in the centre of the arena. A video camera (Casio^c^) connected to a laptop via a fire wire was used to synchronize video and pressure measurements. The video was used to identify the maximal protraction of the inside hindlimb on both reins, at which point pressure data were extracted for analysis.

### 2.6. Kinematics—Two-Dimensional Motion Capture

**Figure 3:**
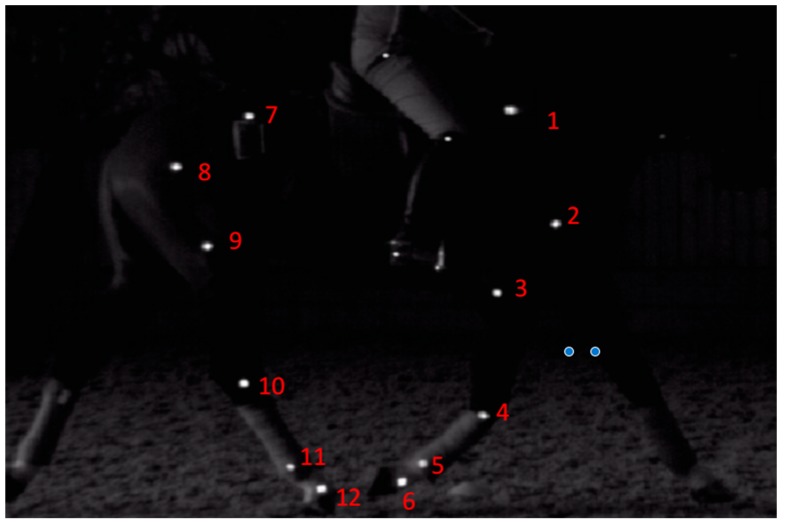
Markers were located over the (1) scapular spine; (2) head of humerus (cranial); (3) lateral condyle of humerus; (4) lateral metacarpal condyles; (5) distal aspect of the metacarpus over the lateral collateral ligament (LCL) of the metacarpophalangeal (MCP) joint; (6) origin of the lateral collateral ligament (LCL) of the distal interphalangeal (DIJ) joint; (7) tuber sacrale; (8) greater trochanter of the femur; (9) lateral condyle of the femur; (10) talus; (11) distal aspect of the metatarsus over the lateral collateral ligament (LCL) of the metatarsophalangeal (MTP) joint; and (12) origin of the lateral collateral ligament (LCL) of the distal interphalangeal (DIJ) joint on both sides of the horse. (Figure 2).

Kinematic data were recorded with a high-speed video camera system, using twenty-four half-sphere skin markers^c^ (30 mm) placed on each horse using double-sided tape. Marker locations have been described elsewhere [27]. In the current study, marker locations were identified by manual palpation of anatomical landmarks identifying joint centres and segment ends. Once located, white skin paint was used to mark each reference point. Markers were located at the following locations: (1) scapular spine; (2) head of humerus (cranial); (3) lateral condyle of humerus; (4) lateral metacarpal condyles; (5) distal aspect of the metacarpus over the lateral collateral ligament (LCL) of the metacarpophalangeal (MCP) joint; (6) origin of the lateral collateral ligament (LCL) of the distal interphalangeal (DIJ) joint; (7) tuber sacrale; (8) greater trochanter of the femur; (9) lateral condyle of the femur; (10) talus; (11) distal aspect of the metatarsus over the lateral collateral ligament (LCL) of the metatarsophalangeal (MTP) joint; and (12) origin of the lateral collateral ligament (LCL) of the distal interphalangeal (DIJ) joint on both sides of the horse. (Figure 3)

Two high speed cameras (Quintic, Four Oaks House, 160 Lichfield Rd, Sutton Coldfield, West Midlands, B742TZ) were positioned at a distance of ten metres from the experiment track, simultaneously capturing the left and right sides of the horse at 400 Hz (spatial resolution 1300 × 400, 400 fps at 10 m distance), with a field of view capturing two complete trot or canter motion cycles. A halogen light was used to illuminate the markers. High speed video data were recorded and downloaded to a laptop (ThinkPad X1, Yoga, Gen4, Lenovo, Redwood 3, Crockford Lane, Chineham Business Park, Basingstoke, RG24 8WQ, UK) and processed using two-dimensional motion capture (Quintic Biomechanics). Described elsewhere [15,24,25,27], automatic marker tracking was used to investigate the following:

- Maximum carpal flexion (palmar angle between (3) lateral condyle of humerus, (4) lateral metacarpal condyles, and (5) distal aspect of the metacarpus over the LCL of the MCP joint).
- Maximum tarsal flexion (angle between lateral condyle of the femur, (10) talus, and (11) distal aspect of the metatarsus over the LCL of the MTP joint) during the swing phase.
- Maximum forelimb fetlock extension during stance (palmar angle between (4) LCL, (5) distal aspect of the metacarpus over the LCL of the MCP joint, and (6) origin of the LCL of the DIP joint)
- Maximum hindlimb fetlock extension during stance (palmar angle between (10) talus, (11) distal aspect of the metatarsus over the LCL of the MTP joint, and (12) origin of the LCL of the DIP joint).

All raw data were smoothed using a Butterworth low-pass filter with a cut off frequency of 10 Hz [28].

### 2.7. Thoracolumbar Epaxial Musculature Dimensions and Height

Thoracolumbar epaxial musculature dimensions were obtained using a new Flexi Curve Ruler (600 mm, Blundell Harling, 9 Albany Road, Granby Industrial Estate, Weymouth, Dorset, DT4 9TH, England), which was shaped around the dorsum, perpendicular to the dorsal midline at three levels of the vertebral column: T10, T13, and T18. Locations (T10, T13, T18) were identified by manual palpation by the same technician. For repeatability, once identified, T10, T13, and T18 were referenced with skin paint. A single SMSQSF performed all measurements, with the horse standing square on a hard, level surface. Measurements of dorsal thoracolumbar body shape of each horse were obtained following the SMS guidelines [17]. The thoracolumbar dimensions (widths at 3 cm and 15 cm ventral to the dorsal midline) were measured at the level of the T10, T13, and T18 [15,26,29,30] prior to ridden exercise and immediately after data had been collected for each saddle (correct, narrow, and wide), and then drawn onto A3 graph paper and the width measured at 3 cm and 15 cm ventral to the dorsal midline. The coefficient of variance was *p* < 0.04 for all measurements using three repeats per measurement.

### 2.8. Data Collection

From the two-dimensional kinematic analysis, data were collected from two consecutive strides with three repeats each, that is, six strides were used for analysis for trot and six strides for canter (three on each rein) for each horse and saddle condition (correct, narrow, and wide).

The outcome parameters for limb kinematics between saddle conditions (correct, narrow, and wide) were as follows:

- Maximum elbow flexion during the swing phase.
- Maximum MCP and MTP hyperextension during stance.
- Maximum carpal flexion during the swing phase.
- Maximum tarsal flexion during the swing phase.

From IMU and pressure distribution, data were matched in relation to movement condition and collected from eleven consecutive strides per repeat totaling a mean ± SD of 33 ± 3 strides, which was used for analysis of the trot and canter on both the left and the right rein for each horse and saddle condition (correct, narrow, and wide).

The outcome parameters for the IMU derived data between saddle conditions (correct, narrow, and wide) were as follows:

- Craniocaudal, vertical, and mediolateral range of motion for T5, T13, T18, and L3 TS.
- Flexion and extension range of motion of T5, T13, T18, and L3 TS.
- Axial rotation range of motion of T5, T13, T18, L3 and TS.
- Lateral bending range of motion of T5, T13, T18, and L3 TS.

The outcome parameters for the pressure distribution between saddle conditions (correct, narrow, and wide) were as follows:

- Pressures beneath the cranial aspect of the saddle (kPa).
- Pressures beneath the caudal aspect of the saddle (kPa).
- Maximum overall force (N).

### 2.9. Statistical Analysis

#### Influence of Speed

As many kinematic parameters are influenced by speed, we tested for differences in speed using a repeated measures analysis of variance (ANOVA) by obtaining stride length and stride time from the two-dimensional analysis for each trial. This was repeated for each saddle condition (correct, narrow, and wide), and no significant differences were found at *p* ≥ 0.05.

Statistical analysis was performed in SPSS (vers. 22, IBM, Armonk, USA). After checking to see if direction (left/right rein) had an effect (all *p* values were found to be ≥0.05), left and right rein data were pooled. A general linear mixed model was used for kinetic and kinematic data, with tree width (correct, narrow, and wide) defined as a fixed factor and horse defined as a random factor. Two general linear models were created; one for trot and one for canter. For all outcome parameters, the significance level was set at *p* ≤ 0.05. A Bonferroni post hoc analysis was carried out to determine differences between pairs of conditions (correct, narrow, and wide). Instead of applying the Bonferoni correction on the significance level, alpha, this study reported the Bonferroni adjusted *p*-values (*p*-values based on Fisher’s Least Significant Difference (LSD) multiplied by the number of comparisons done). This allowed us to assess the significance of our results (based on Bonferroni adjusted *p*-values) to the traditional alpha of 5%, without increasing type II errors.

## 3. Results

### 3.1. Horse Inclusion

One horse was excluded because his thoracolumbar dimensions were too narrow, resulting in a narrow saddle not being able to be fitted. From the subjective veterinary assessment, all horses were deemed fit to perform. From the objective movement asymmetry measures in trot on the straight, horses had (mean ± SD) asymmetry values: HDmin, 1.47 ± 2.21 mm and HDmax 1.55 ± 2.72 mm, Pelvis Min_Diff_ 2.35 ± 2.80 mm and Pelvis Max_Diff_ 2.0 ± 2.12 mm, and HHD 1.48 ± 4.98 mm.

### 3.2. Thoracolumbar Kinematics

#### 3.2.1. Trot—Rotational and Translational Movement

**Figure 4:**
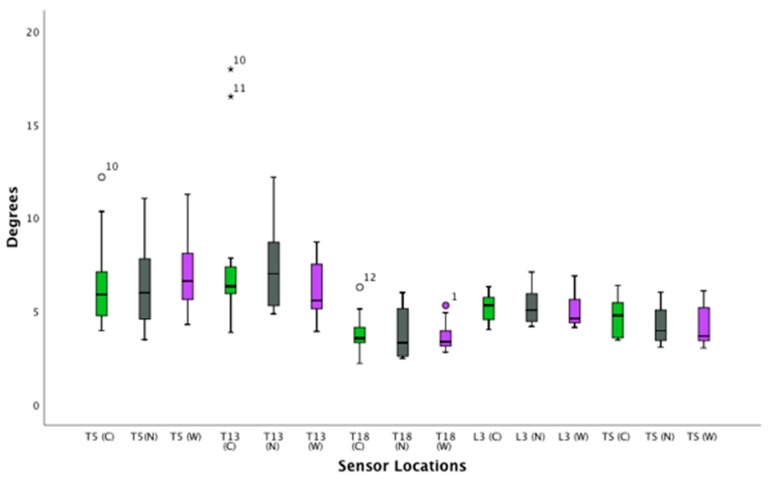
Flexion and extension of the thoracolumbar spine in trot. Boxplots displaying flexion-extension of the thoracolumbar spine in thirteen horses while trotting for the three saddle conditions: correct saddle (green), narrow saddle (grey), and wide saddle (purple). The central line represents the median; the box represents the 25th and 75th percentiles; and the whiskers represent the maxima and minima not considered to be outliers. ⚬ represents outliers and * represents extreme outliers. Post hoc analysis showed an increase in flexion-extension at T13 between the correct and narrow saddle widths (4%, *p* = 0.04).

**Figure 5:**
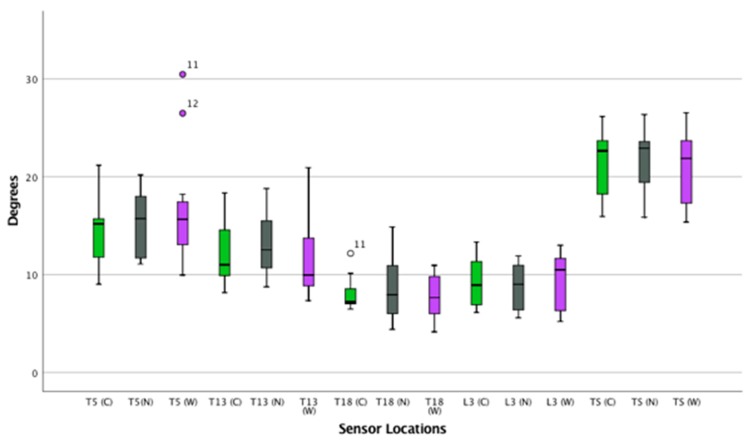
Axial rotation of the thoracolumbar spine in trot. Boxplot displaying axial rotation of the thoracolumbar spine in thirteen horses while trotting for the three saddle conditions: correct saddle (green), narrow saddle (grey), and wide saddle (purple). The central line represents the median; the box represents the 25th and 75th percentiles; and the whiskers represent the maxima and minima not considered to be outliers. ⚬ represents outliers. Post hoc analysis showed an increase in axial rotation at T13 between the correct and narrow saddle widths (3%, *p* = 0.04).

**Figure 6:**
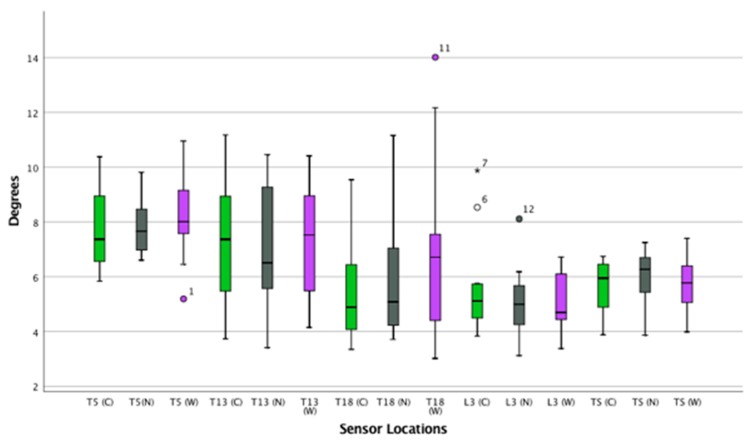
Lateral bending of the thoracolumbar spin in trot. Boxplots displaying lateral bending of the thoracolumbar spine while trotting in thirteen horses for the three saddle conditions: correct saddle (green), narrow saddle (grey), and wide saddle (purple). The central line represents the median; the box represents the 25th and 75th percentiles; and the whiskers represent the maxima and minima not considered to be outliers. ⚬ represents outliers and * represents extreme outliers.

(Supplementary Tables S1 and S2) (Figure 4, Figure 5 and Figure 6)

T5

Craniocaudal range of motion (ROM) differed according to saddle widths (*p* = 0.02). Post hoc analysis showed an increase in craniocaudal ROM between the correct and wide saddle (6.2%, *p* = 0.01). Post hoc analysis showed no significant differences between the correct and narrow saddle widths (*p* ≥ 0.05). 

T13

Flexion-extension differed according to saddle widths (*p* = 0.03). Post hoc analysis showed an increase in flexion-extension between the correct and narrow saddle widths (4%, *p* = 0.04). Post hoc analysis showed no significant differences between the correct and wide saddle widths (*p* ≥ 0.05).

Axial rotation differed according to saddle widths (*p* = 0.03). Post hoc analysis showed an increase in axial rotation between the correct and narrow saddle widths (3%, *p* = 0.04). Post hoc analysis showed no significant differences between the correct and wide saddle widths (*p* ≥ 0.05).

Vertical movement differed according to saddle widths (*p* = 0.003). Post hoc analysis showed decreased vertical movement between the correct and narrow saddle widths (6%, *p* = 0.004). Post hoc analysis showed no significant differences between the correct and wide saddle widths (*p* ≥ 0.05).

Mediolateral ROM differed according to saddle widths (*p* = 0.001). Post hoc analysis showed decreased vertical movement between the correct and wide saddle widths (6%, *p* = 0.02) and increased vertical movement between the correct and narrow saddle widths (8%, *p* = 0.004).

T18

No changes in any movement parameters were found; all *p* ≥ 0.05

L3

Axial rotation differed according to saddle widths (*p* = 0.03) Bonferroni corrected post hoc analysis did not identify any pairwise significant differences (all *p* ≥ 0.14)

TS

No changes in any movement parameters were found; *p* > 0.05

#### 3.2.2. Canter—Rotational and Translation Movement 

**Figure 7:**
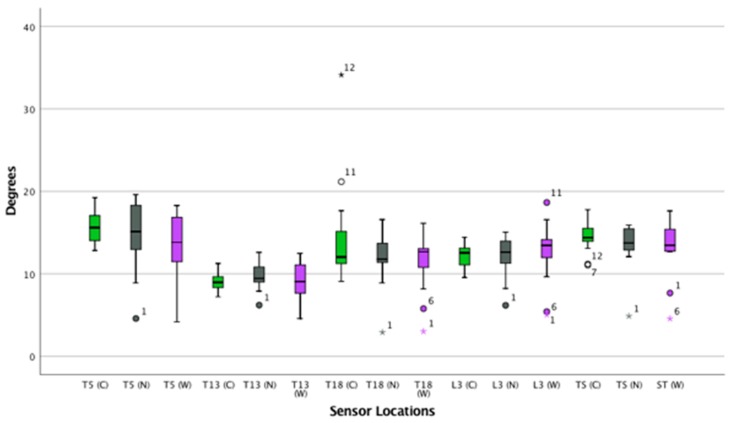
Flexion and extension of the thoracolumbar spine in canter. Boxplots displaying flexion-extension of the thoracolumbar spine in thirteen horses while cantering for the three saddle conditions: correct saddle (green), narrow saddle (grey), and wide saddle (purple). The central line represents the median; the box represents the 25th and 75th percentiles; and the whiskers represent the maxima and minima not considered to be outliers. ⚬ represents outliers and * represents extreme outliers. Post hoc analysis showed a decrease in flexion-extension at T18 between the wide and the correct saddle widths (5%, *p* = 0.04).

**Figure 8:**
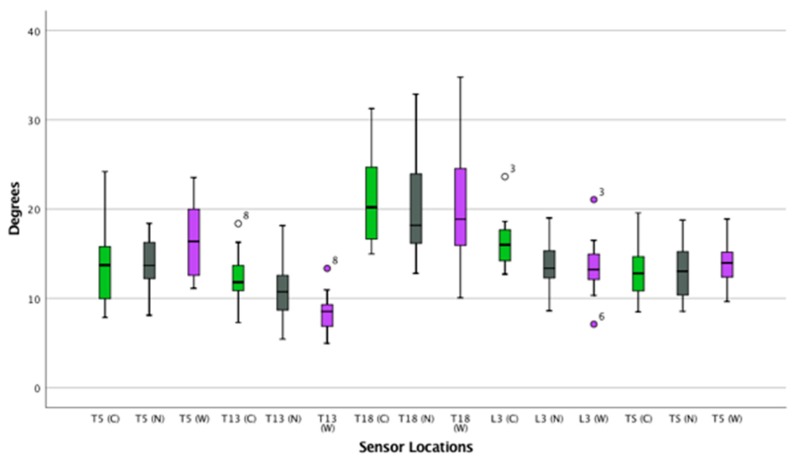
Axial rotation on the thoracolumbar spine while cantering. Boxplots displaying axial rotation of the thoracolumbar spine in thirteen horses whilst cantering for the three saddle conditions: correct saddle (green), narrow saddle (grey), and wide saddle (purple). The central line represents the median; the box represents the 25th and 75th percentiles; and the whiskers represent the maxima and minima not considered to be outliers. ⚬ represents outliers. Post hoc analysis showed an increase in axial rotation at T5 between the correct and wide saddle (1%, *p* = 0.03), a decrease in axial rotation between the correct and wide saddle (5%, *p* = 0.04) at T13, and a decrease in axial rotation at L3 between the correct and wide saddle width (5%, *p* = 0.03).

**Figure 9:**
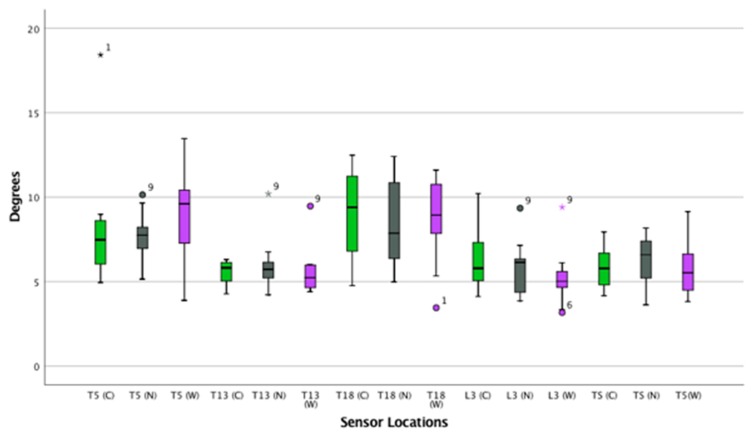
Lateral bending of the thoracolumbar spine while cantering. Boxplots displaying lateral bending of the thoracolumbar spine in thirteen horses while cantering for the three saddle conditions: correct saddle (green), narrow saddle (grey), and wide saddle (purple). The central line represents the median; the box represents the 25th and 75th percentiles; and the whiskers represent the maxima and minima not considered to be outliers. ⚬ represents outliers and * represents extreme outliers.

(Supplementary Tables S3 and S4) (Figure 7, Figure 8 and Figure 9)

T5

Axial rotation differed according to saddle widths (*p* = 0.02). Post hoc analysis showed an increase in axial rotation between the correct and wide saddle (1%, *p* = 0.03). Post hoc analysis showed no significant differences between the correct and narrow saddle widths (*p* ≥ 0.05).

T13

Axial rotation differed according to saddle widths (*p* = 0.01). Post hoc analysis showed a decrease in axial rotation between the correct and wide saddle (5%, *p* = 0.04).

Vertical movement of T13 was different between saddle widths (*p* ≤ 0.05). Post hoc analysis showed no significant differences between saddle width conditions (*p* ≥ 0.05).

T18

Flexion-extension differed according to saddle widths (*p* = 0.03). Post hoc analysis showed a decrease in flexion-extension between the wide and the correct saddle widths (4%, *p* = 0.04). Post hoc analysis showed no significant differences between the correct and narrow saddle widths (*p* ≥ 0.05).

Lateral bending was affected by saddle widths (*p* = 0.03). Post hoc analysis showed no significant differences between saddle width conditions (*p* ≥ 0.14).

In the mediolateral direction, the ROM of T18 differed according to saddle widths (*p* ≤ 0.05). Post hoc analysis showed no significant differences between saddle width conditions (*p* ≥ 0.05).

L3

Axial rotation differed according to saddle width for both the narrow and wide saddle (*p* = 0.007). Post hoc analysis showed a decrease in axial rotation between the correct and wide saddle width (5%, *p* = 0.03) and no significant difference between the correct and narrow saddle (*p* = 0.10).

In the mediolateral direction, the L3 ROM differed according to saddle widths (*p* ≤ 0.05). Bonferroni corrected post hoc analysis did not identify any pairwise significant differences (all *p* ≥ 0.14)

#### 3.2.3. Trot—Rotational and Translational Movement 

(Supplementary Tables S1 and S2)

TS

No changes in any movement parameters were found; all *p* > 0.05.

### 3.3. Kinetic Data—Pressure Distribution

#### 3.3.1. Trot

Peak pressures (kPa) differed between saddles in the cranial region of the saddle (*p* = 0.003). Post hoc analysis showed an increase in peak pressures (kPa) in the cranial region of the saddle between the correct and wide saddle widths (9%, *p* = 0.002). Post hoc analysis showed no significant differences between peak pressures (kPa) in the cranial region of the correct and narrow saddle widths (*p* ≥ 0.05).

Peak pressures (kPa) differed between saddles in the caudal region of the saddle (*p* = 0.003). Post hoc analysis showed an increase in peak pressures (kPa) in the caudal region of the saddle between the correct and narrow saddle widths (14%, *p* = 0.01) and narrow and wide saddle widths (16%, *p* = 0.01).

The overall force (N) in the cranial region differed between saddles, but was not found to be significant (*p* = 0.11). Overall, force differed in the caudal region between saddle widths (*p* = 0.02). Post hoc analysis showed an increase in overall force (N) in the caudal region of the saddle between the wide and narrow saddle widths (8%, *p* = 0.002) (Table 2).

#### 3.3.2. Canter

Peak pressures (kPa) in thirteen horses differed between saddles in the cranial region of the saddle (*p* = 0.04). Post hoc analysis showed an increase in peak pressures (kPa) in the cranial region of the saddle between the correct and wide saddle widths (5%, *p* = 0.003). Post hoc analysis showed no significant differences between peak pressures (kPa) in the cranial region of the correct and narrow saddle widths (*p* ≥ 0.05).

The overall force (N) in the cranial region differed between saddles (*p* = 0.001). Post hoc analysis showed a decrease in overall force (N) in the cranial region of the saddle between the correct and narrow saddle widths (9%, *p* = 0.002) (Table 3). Post hoc analysis showed no significant differences between overall force (N) in the cranial region of the correct and wide saddle widths (*p* ≥ 0.05).

### 3.4. Kinematics—Two-Dimensional Motion Capture

Maximum tarsal flexion differed between saddle widths (*p* = 0.03). Post hoc analysis showed an increase in maximum tarsal flexion between the narrow and wide saddle widths (3%, *p* = 0.01). All other parameters were *p* ≥ 0.05 (Table 4).

### 3.5. Thoracolumbar Epaxial Musculature Profiles

Thoracolumbar epaxial musculature profiles differed between saddle widths (*p* = 0.03). Post hoc analysis showed a decrease at the level of T13 (3 cm ventral to the dorsal midline) between the correct and wide saddle widths (14%, *p* = 0.02). Post hoc analysis showed no significant differences between the narrow and correct saddle widths (*p* ≥ 0.05).

Thoracolumbar epaxial musculature profiles differed between saddle widths (*p* = 0.03). Post hoc analysis showed a decrease at the level of T18 (3 cm ventral to the dorsal midline) between the correct and narrow saddle widths (4%, *p* = 0.04). Post hoc analysis showed no significant differences between the wide and correct saddle widths (*p* ≥ 0.05) (Table 5).

## 4. Discussion

**Figure 10:**
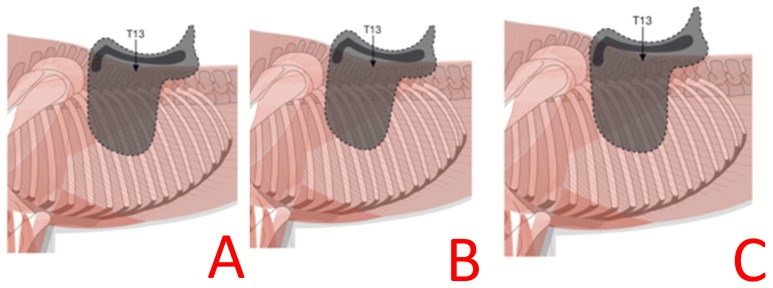
Illustrating the orientation of the saddle tree for the three saddle width conditions. A = correct, B = narrow, and C = wide saddle widths. In the correct saddle width (**A**), the tree lies parallel to the horse’s back. As a result of the narrow saddle (**B**), the cranial region of the saddle is pitched dorsally. In contrast, in the wide saddle (**C**), the cranial region of the saddle is pitched ventrally.

This experiment quantified thoracolumbar kinematics during ridden exercise in trot and canter locomotion with the use of inertial measuring units. Following published protocols [19], this study positioned IMUs along the thoracolumbar spine beneath the saddle similar to previous methods [19,31,32,33,34]. In partial support of our hypothesis, in both trot and canter, a saddle that was fitted wide or narrow resulted in changes in thoracolumbar kinematics when compared with a saddle that was fitted following industry guidelines. It has been reported that, despite a cohort of qualified saddle fitters receiving the same training, determining tree width was challenging and agreement was ‘only’ slight (57%, k = 0.12) [14]. The aim of this experiment was to quantify the effect that saddle width has on equine locomotion, thoracolumbar kinematics, epaxial musculature dimensions, and saddle pressure distribution. In accordance with our hypothesis, in trot, smaller flexion-extension values for T13 were found when ridden in the narrow saddle. The ROM of T13 increased in the mediolateral direction and decreased in the vertical direction when ridden in a narrow saddle compared with a correctly fitted saddle. It is proposed that the changes reported here are as a result of the positioning of the saddle (Figure 10) and the resultant change in pressure distribution. In the narrow saddle, larger values for axial rotation were found for T13. The narrow saddle has four points of contact, which results in the mid region of the saddle not being in contact with the horse. It is speculated that, as a result of this area having lower saddle pressures, this in some way allows for increased axial rotation. This idea warrants further investigation. Although axial rotation increased at T13, decreased flexion-extension was observed. A decrease thoracolumbar ROM has been reported in horses with back dysfunction [35]; therefore, it is speculated that incorrect saddle width (narrow) induces a vertebral movement pattern similar to that observed in horses with back dysfunction, hence the importance of correct saddle fit.

The cranial region of the thoracic spine (T10–T13) is of great interest; it is where the rider’s centre of mass aligns approximately above the horse’s centre of mass. It is the area where the saddle is at its narrowest and it is also a region that has been reported to be associated with muscle activity [36] and improved gait features when saddle pressure in this region was reduced after modified saddle design [15,26]. Increased amplitudes have been reported in the dorsoventral ROM occurring at T13 compared with T18 and L3 in non-lame horses trotting in a straight line [37]. The association between lameness and thoracolumbosacral ROM has been investigated, and it was reported that, after diagnostic analgesia, there was an increase in ROM in flexion-extension at T13, axial rotation at T13, T18 L3, and in lateral–lateral ROM at L3 [4]. The effect of tree width on equine locomotion was quantified with the rider sitting to the trot throughout each motion cycle. It is appreciated that the findings may not be reflective of rising trot and future work should quantify all riding positions [33,38,39,40,41,42], including rising ‘on the wrong diagonal’, which has recently been shown to elicit the largest amount of movement asymmetry in the horse [43].

There is limited evidence on thoracolumbar kinematics in canter during ridden exercise. In this experiment, it was observed that the wide saddle reduced flexion-extension at T18, which could be the result of the saddles’ balance and stability. Additionally, in the wide saddle, axial rotation was increased at T5 and decreased at T13 and L3. These findings would be in contrast to the belief by some saddle fitters that increased tree width allows for increased spinal kinematics. In the wide saddle, the cranial aspect of the saddle is pitched ventrally and it is speculated that this positioning (and increased pressures) limits the spinal range of motion. Our values are slightly higher for flexion-extension and axial rotation than those reported for horses cantering on a treadmill [10]. This could be explained by the effect that the saddle and rider have on thoracolumbar kinematics [44], as well as the fact that this study cantered horses over ground versus on a treadmill [45].

Further support that tree width affects equine locomotion arises from the saddle pressure distribution. In accordance with the hypothesis, in this group of horses, changes in saddle pressures and location of pressure distribution when fitted with a wide and narrow saddle were found. The findings reported here are in accordance with those reported elsewhere [3]. In a wide saddle, the underneath of the saddle, and consequently the tree, pitches close to the horse’s spine. As a consequence, the increased width of the saddle allows the cranial aspect of the saddle to displace ventrally, creating insufficient room for unrestricted lateroflexion of the thoracic spine [3]. This is also supported by our IMU findings, as smaller lateral bending values were found for T13, T18, and L3 in the wide saddle. Although the English saddle has been shown to effectively distribute saddle pressures, the orientation of the wide saddle (cranial aspect pitched ventrally) will likely affect the performance of the saddle in distributing the pressures effectively [46]. The results presented here indicate areas of high pressure in the region of T10–T13. Peak pressures in canter (>35 kPa) were higher than those reported to cause skin ischemia and ulceration [47]. Dry spots after exercise can be an indicator of high pressure [48]. There were no signs of dry spots in the current study, which could be because the exercise test performed in the current study was of insufficient intensity to induce a sweating response.

Similar to previous findings [3], the results being presented here indicate that in the wide saddle, areas of high pressures were close to the midline of the equine spine in the region T10–T13 in both trot and canter. Furthermore, from a visual subjective observation, the wide saddle was unstable with excessive dorsoventral displacement of the caudal region of the saddle, which increased further when cantering. This displacement of the saddle is likely influenced by the kinematics of the thoracolumbar spine when cantering [10].

When riding with the narrow saddle, the cranial part of the saddle is pitched dorsally (front rising up) and, as a result, the seat of the saddle is not parallel to the horse’s back, causing the saddle to rock backwards [3,7]. Saddles that have four points of contact are defined by saddle fitters as bridging. Dynamically, the four points of contact may be better represented as a rocking motion cranial to caudal and side to side. In either case, reduced contact beneath the middle region of the saddle can be seen. Focal pressures can occur in the caudal aspect of the saddle, which have the potential to induce concavities in the epaxial musculature [7]. In the current experiment, areas of high pressures were found beneath the caudal aspect of the narrow saddle, which was associated with concavities in the epaxial musculature of T18.

Finally, further support that saddle width has an effect on the thoracolumbar spine arises from our thoracolumbar dimensions. The thoracolumbar musculature has been reported to change over a year as a result of changes in body weight, seasonal variations, and saddle fit [30], as well as after thirty minutes of exercise when horses are worked correctly in a well fitted saddle [29], and also after modifications of the saddle resulting in reduced saddle pressures in the thoracic region [15,26]. In the current study, smaller epaxial musculature dimensions were found at T13 after ridden exercise in the wide saddle. In contrast, in the narrow saddle, reduced musculature dimensions were found at T18. Reduced musculature dimensions can be interpreted as concavities in the musculature. Musculature concavities are thought to occur as a result of focal pressures. This seems likely as, here, areas of high saddle pressures were found in the region of T10–T13 for the wide saddle and T18 for the narrow saddle. The sensors remained on the horse throughout; however, for the site of T13 and T18, the sensor was removed to allow for thoracolumbar measurements to be taken. Both areas were identified with skin paint. We are confident, therefore, that both the sensor positioning and thoracolumbar measurements were consistent between measurements, but this should be considered when interpreting the findings [49].

Although differences have been reported in this study, the authors appreciate that this study is limited by the number and type of horses used and that only two riders were used. The use of the Pliance pressure mat beneath the saddle may have influenced saddle fit, although this was consistent for all conditions. The effect that the Pliance mat may have on saddle fit should be considered when interpreting the results. The use of a girth with elastic on one side may have contributed to saddle instability and, if the study were to be repeated, then a non-elasticated girth should be considered. The changes observed are immediate and, although statistically significant (*p* ≤ 0.05), are in some cases of a small magnitude. Although some of the changes reported are small, it is speculated over time that these changes may have an effect on the locomotor apparatus of the horse; hence, a longitudinal study is warranted. Studies have reported changes in equine locomotion when pressures beneath a saddle [15], bridle [25], and girth [24] have been reduced; therefore, it is speculated that in the current study, riding in a saddle that is too wide or narrow, resulting in areas of high pressures in either the cranial or caudal regions, will have a deleterious effect on the equine locomotor apparatus and compromise back health and function. It is appreciated that this study has reported one aspect associated with saddle fitting; therefore, future studies should attempt to quantify other saddle fitting parameters.

## 5. Conclusions

Correct saddle fit is essential to provide unhindered back function and optimise the interaction between the horse and rider dyad. This study has investigated the effect of tree width on thoracolumbar kinematics, limb kinematics, saddle pressure distribution, and thoracolumbar dimensions. A saddle that is fitted too wide or too narrow resulted in altered pressure distribution and, in some regions, changes in thoracolumbar spinal kinematics. Here, immediate changes were investigated and a longitudinal study would be useful in determining the long-term effect of varying tree widths. Horse owners should consider the effects that tree width has on the horse and seek regular professional advice to ensure optimal saddle fit. It is hoped that the findings from this study and those presented elsewhere [3,50] will be used to further the understanding among saddle fitters about the importance of fitting saddles of a correct width.

## Figures and Tables

**Table 1:** Saddle widths for each horse and order of testing. Correct width was determined by five qualified saddle fitters and an objective measuring system. The number (1, 2, 3) refers to the order in which the saddles were assessed.

Horse ID	Correct Saddle Fit	Wide Saddle Fit	Narrow Saddle Fit
1	Medium 1	Wide 2	Narrow 3
2	Wide 3	Ex Wide 1	Medium 2
3	Medium 2	Wide 3	Narrow 1
4	Medium 1	Wide 2	Narrow 3
5	Medium 3	Wide 1	Narrow 2
6	Medium 2	Wide 3	Narrow 1
7	Medium 1	Wide 2	Narrow 3
8	Medium 3	Wide 1	Narrow 2
9	Medium 2	Wide 3	Narrow 1
10	Medium 1	Wide 2	Narrow 3
11	Medium 3	Wide 1	Narrow 2
12	Medium 2	Wide 3	Narrow 1
13	Medium 1	Wide 2	Narrow 3

**Table 2:** Saddle pressure distribution data collected from thirty-three strides from beneath the saddle during trot for all saddle width conditions. Left and right rein data pooled with a significance level set at *p* ≤ 0.05 with a Bonferroni post hoc adjustment to determine differences between conditions, with only significant post hoc results being presented (Bonferroni adjusted alpha 5%).

Location of Pressure	Correct Mean ± SD	Wide Mean ± SD	Narrow Mean ± SD	*p*-Value	Post Hoc: Bonferroni
Cranial Region Peak Pressure (kPa)	36.3 ± 6.9	39.4 ± 7.1	36.3 ± 7.2	0.003	narrow-wide, *p* = 0.002
Caudal Region Peak Pressure (kPa)	26.5 ± 2.7	25.9 ± 3.6	30.2 ± 2.3	0.003	correct-narrow, *p* = 0.01 narrow-wide, *p* = 0.01
Cranial Region Maximum Overall Force (N)	803.3 ± 165.9	840.6 ± 158.3	777.8 ± 168.5	0.11	-
Caudal Region Maximum Overall Force (N)	660.0 ± 145.5	641.3 ± 144.8	713.1 ± 160.8	0.02	narrow-wide, *p* = 0.002
Overall Force (N)	1463.4 ± 310.8	1482.0 ± 298.2	1490.0 ± 329.3	-	-
Cranial Region Mean Pressure (kPa)	14.01.5	14.5 ± 1.3	12.8 ± 2.2	0.0001	correct-wide, *p* = 0.03 narrow-wide, *p* = 0.001
Caudal Region Mean Pressure (kPa)	9.1 ± 0.3	11.9 ± 3.1	11.0 ± 2.6	0.001	correct-narrow, *p* = 0.002 correct-wide, *p* = 0.02

**Table 3:** Saddle pressure distribution data collected from thirty-three strides from beneath the saddle during canter for all saddle width conditions from thirteen horses. Left and right rein data pooled with a significance level set at *p* ≤ 0.05 with a post hoc adjustment using Bonferroni to determine differences between conditions, with only significant post hoc results being presented (Bonferroni adjusted alpha 5%).

Location of Pressure	Correct Mean ± SD	Wide Mean ± SD	Narrow Mean ± SD	*p*-Value	Post hoc: Bonferroni
Cranial Region Peak Pressure (kPa)	44.43 ± 8.85	46.06 ± 8.71	42.36 ± 7.46	*p* = 0.04	correct-wide *p* = 0.003
Caudal Region Peak Pressure (kPa)	31.07 ± 2.86	31.07 ± 3.38	30.05 ± 3.67	*p* = 0.08	-
Cranial Region Maximum Overall Force (N)	832.16 ± 107.46	819.15 ± 109.63	759.46 ± 113.40	*p* = 0.001	correct-narrow *p* = 0.002
Caudal Region Maximum Overall Force (N)	627.39 ± 66.72	611.01 ± 42.86	647.39 ± 65.92	*p* = 0.04	-
Cranial Region Mean Pressure (kPa)	15.48 ± 2.31	14.82 ± 2.06	14.82 ± 2.06	*p* = 1.3	-
Caudal Region Mean Pressure (kPa)	11.42 ± 0.78	11.26 ± 0.73	11.45 ± 1.12	*p* = 1.32	-

**Table 4:** Simultaneous motion capture providing kinematic data collected for the left and right side while trotting for all conditions in thirteen horses. Left and right rein data pooled with a significance level set at *p* ≤ 0.05 with a Bonferroni post hoc adjustment to determine differences between conditions, with only significant post hoc results being presented (Bonferroni adjusted alpha 5%).

Kinematic Parameters	Correct Mean ± SD (°)	Wide Mean ± SD (°)	Narrow Mean ± SD (°)	*p*-Value	Post hoc: Bonferroni
Elbow Flexion (swing phase)	103.9 ± 10.8	101.4 ± 3.6	100.0 ± 4.5	0.39	-
Maximum carpal flexion (swing phase)	92.3 ± 7.9	91.4 ± 6.7	89.8 ± 6.7	0.03	-
Maximum front fetlock hyperextension—(stance phase)	111.2 ± 7.3	113.7 ± 11.4	109.6 ± 6.3	0.31	-
Maximum hip flexion (swing phase)	99.8 ± 12.9	100.0 ± 11.5	101.9 ± 8.1	0.62	-
Maximum tarsal flexion (swing phase)	119.9 ± 10.4	119.7 ± 9.3	116.7 ± 9.0	0.03	narrow-wide, *p* = 0.01
Maximum hind fetlock hyperextension (stance phase)	113.7 ± 6.0	113.5 ± 7.4	114.5 ± 5.7	0.66	-

**Table 5:** Thoracolumbar epaxial musculature profiles at the tenth thoracic vertebra (T10), thirteenth thoracic vertebra (T13), and eighteenth thoracic vertebra (T18) for each condition measured 3 cm and 5 cm ventral from the dorsal midline for thirteen horses. Significance level was set at *p* ≤ 0.05 with a Bonferroni post hoc adjustment to determine differences between conditions, with only significant post hoc results being presented (Bonferroni adjusted alpha 5%). A decrease in epaxial musculature profiles at T13 was found after ridden exercise in the wide saddle (14%, *p* = 0.02) and at the site of T18 after ridden exercise in the narrow saddle (4%, *p* = 0.04).

Measuring Sites on the Thoracic Region	Correct Mean ± SD (cm)	Wide Mean ± SD (cm)	Narrow Mean ± SD (cm)	*p*-Value	Post hoc: Bonferroni
T10 (3 cm)	8.14 ± 1.6	8.2 ± 1.1	8.0 ±1.0.	0.47	
T10 (15 cm)	30.9 ± 1.9	32.0 ± 2.9	31.4 ± 1.9	0.07	
T13 (3 cm)	13.6 ± 1.7	11.2 ± 1.7	13.7 ± 1.9	0.03	Correct—wide, *p* = 0.02
T13 (15 cm)	46.2 ± 1.6	46.2 ± 1.9	45.7 ± 1.9	0.24	
T18 (3 cm)	21.3 ± 2.5	21.3 ± 2.6	19.3 ± 2.2	0.03	Correct—narrow, *p* = 0.04
T18 (15 cm)	52.7 ± 21.1	52.3 ± 2.3	52.9 ± 2.0	0.44	

## Supplementary Materials

The following are available online at https://www.mdpi.com/2076-2615/9/10/842/s1, Table S1: Translational IMU kinematic data whilst trotting for each saddle condition, correct, narrow and wide. Significance level set at *p* ≤ 0.05 with a Bonferroni post hoc correction to determine pairwise significance between conditions with only significant post hoc results being presented (Bonferroni adjusted alpha 5%). (DV = dorsoventral ROM, ML = mediolateral ROM, CC = craniocaudal ROM), Table S2: Rotational IMU kinematic data whilst trotting for each saddle condition, correct, narrow and wide. Significance level set at *p* ≤ 0.05 with a Bonferroni post hoc correction to determine pairwise significance between conditions with only significant post hoc results being presented (Bonferroni adjusted alpha 5%). (FE = Flexion-Extension, AR = Axial Rotation, LB = Lateral Bending), Table S3: Displaying IMU kinematic data whilst cantering for each saddle condition, correct, narrow and wide. Significance level set at *p* ≤ 0.05 a Bonferroni post hoc correction to determine pairwise significance between conditions with only significant post hoc results being presented (Bonferroni adjusted alpha 5%). (DV = dorsoventral ROM, ML= mediolateral ROM, CC = craniocaudal ROM), Table S4: Displaying IMU kinematic data whilst cantering for each saddle condition, correct, narrow and wide. Significance level set at *p* ≤ 0.05 with a Bonferroni post hoc correction to determine pairwise significance between conditions with only significant post hoc results being presented (Bonferroni adjusted alpha 5%). (FE = flexion-extension, AR = Axial Rotation, LB = Lateral Bending).
