# Supplementary material for: The Effect of Tree Width on Thoracolumbar and Limb Kinematics, Saddle Pressure Distribution, and Thoracolumbar Dimensions in Sports Horses in Trot and Canter
Source: Animals (Basel). 2019 Oct 21;9(10):842. doi: 10.3390/ani9100842 (PMC6827167; doi:10.3390/ani9100842)
Supplement: Supplementary file 1 [file animals-09-00842-s001.pdf]

Supplementary Material

Table S1. - Translational IMU kinematic data whilst trotting for each saddle condition, correct, narrow and wide. Significance level set at $p \leq 0.05$ with a Bonferroni post hoc correction to determine pairwise significance between conditions with only significant post hoc results being presented (Bonferroni adjusted alpha 5%). (DV = dorsoventral ROM, ML= mediolateral ROM, CC = craniocaudal ROM).

	Saddle Condition	**T5 Mean ± SD (mm)**	**T13 Mean ± SD (mm)**	**T18 Mean ± SD (mm)**	**L3 Mean ± SD (mm)**	**TS Mean ± SD (mm)**
DV	Correct	71.86 ± 10.3	95.3 ± 9.3	97.0 ± 11.1	91.7 ± 10.7	86.5 ± 11.1
DV	Narrow	72.91 ± 11.3	90.9 ± 11.7	96.0 ± 10.6	92.7 ± 12.6	87.2 ± 1.8
DV	Wide	73.00 ± 1.1	92.6 ± 11.6	95.0 ± 11.2	91.2 ± 11.4	86.4 ± 11.1
P-value		0.62	**0.003**	0.62	0.35	0.53
Bonferroni post hoc P-value		-	correct – narrow, 0.004	-	-	-
ML	Correct	28.82 ± 4.8	19.6 ± 5.4	29.6 ± 8.5	26.8 ± 5.0	37.5 ± 7.9
ML	Narrow	27.5 ± 4.9	21.0 ± 5.6	28.5 ± 9.1	28.9 ±7.2	37.2 ± 8.3
ML	Wide	29.2 ± 7.2	17.8 ± 5.2	28.7 ± 8.8	29.9 ± 6.0	36.5 ± 8.1
P-value		0.16	**0.001**	0.78	0.08	0.48
Bonferroni post hoc P-value		-	correct – wide, 0.02. correct – narrow, 0.004	-	-	-
CC	Correct	32.9 ± 7.4	27.6 ± 4.6	18.3 ±4.4	14.2 ± 3.0	15.1 ± 3.2
CC	Narrow	31.9 ± 7.3	28.5 ±5.1	18.5 ± 4.3	14.3 ± 3.7	15.0 ± 3.8
CC	Wide	34.0 ± 7.5	28.8 ± 4.1	16.6 ± 3.2	14.2 ± 4.0	15.1 ± 3.3
P-value		**0.02**	0.31	0.08	0.98	0.97
Bonferroni post hoc P-value		narrow – wide, 0.01	-	-	-	-

Table S2. –Rotational IMU kinematic data whilst trotting for each saddle condition, correct, narrow and wide. Significance level set at p <0.05 with a Bonferroni post hoc correction to determine pairwise significance between conditions with only significant post hoc results being presented (Bonferroni adjusted alpha 5%). (FE=Flexion-Extension, AR = Axial Rotation, LB = Lateral Bending).

	Saddle Condition	**T5 Mean ± SD (°)**	**T13 Mean ± SD (°)**	**T18 Mean ± SD (°)**	**L3 Mean ± SD (°)**	**TS Mean ± SD (°)**
FE	Correct	6.43 ± 2.3	7.50 ± 3.3	3.79 ±1.0	5.20 ± 0.7	4.25 ± 1.0
FE	Narrow	6.05 ± 2.3	5.19 ± 2.4	2.96 ± 1.2	4.31 ± 0.8	3.10 ± 1.2
FE	Wide	6.93 ± 1.8	6.08 ± 1.4	3.62 ± 0.7	5.00 ± 0.9	4.18 ± 0.7
P-value		0.81	**0.03**	0.42	0.67	0.66
Bonferroni post hoc P-value		-	correct – narrow, 0.04	-	-	-
AR	Correct	16.94 ± 3.3	11.64 ± 3.2	7.69 ± 1.0	9.41 ± 2.4	20.94 ± 0.9
AR	Narrow	15.27 ± 3.1	13.14 ± 2.9	8.57 ± 1.2	8.79 ± 2.4	21.66 ± 0.9
AR	Wide	15.60 ± 5.6	10.09 ± 4.0	6.96 ± 0.7	8.42 ± 2.7	16.16 ± 1
P-value		**0.04**	**0.03**	0.88	**0.03**	0.08
Bonferroni post hoc P-value		-	correct – narrow, 0.04	-	-	-
LB	Correct	8.21 ± 1.4	7.2 ± 2.0	5.9 ± 1.9	5.06 ± 1.7	5.7 ± 1.9
LB	Narrow	7.82 ± 0.9	7.1 ± 2.0	5.9 ± 2.2	5.05 ± 1.2	5.9 ± 2.2
LB	Wide	6.67 ±1.4	6.2 ± 2.1	6.8 ± 3.22	4.06 ± 1.0	4.3 ± 0.9
P-value		0.22	0.54	0.22	0.89	0.33
Bonferroni post hoc P-value		-	-	-	-	-

Table S3 – Displaying IMU kinematic data whilst cantering for each saddle condition, correct, narrow and wide. Significance level set at $p \leq 0.05$ a Bonferroni post hoc correction to determine pairwise significance between conditions with only significant post hoc results being presented (Bonferroni adjusted alpha 5%). (DV = dorsoventral ROM, ML= mediolateral ROM, CC = craniocaudal ROM).

	Saddle Condition	**T5 Mean ± SD**	**T13 Mean ± SD**	**T18 Mean ± SD**	**L3 Mean ± SD**	**TS Mean ± SD**

		(mm)	(mm)	(mm)	(mm)	(mm)
DV	Correct	185.4 ± 18.8	216.3 ± 12.8	207.1 ± 23.4	196.5 ± 37.0	193.5 ± 21.0
DV	Narrow	167.0 ± 36.7	200.0 ± 42.1	194.9 ± 40.2	190.7 ± 49.5	181.3 ± 36.7
DV	Wide	165.3 ± 49.7	186.42 ± 49.8	192.9 ± 49.9	188.5 ± 49.5	178.7 ± 49.3
P- value		0.18	0.05	0.43	0.73	0.30
Bonferroni post hoc P - value		-	-	-	-	-
ML	Correct	51.3 ± 16.6	51.2 ± 7.67	73.8 ± 26.8	69.0 ± 22.9	55.6 ± 10.3
ML	Narrow	43.7 ± 10.2	44.5 ± 11.41	57.0 ± 13.63	54.0 ± 10.4	53.4 ± 10.0
ML	Wide	44.7 ± 12.7	58.4 ± 37.6	54.3 ± 15.03	55.0 ± 15.6	55.1 ± 12.9
P - value		0.31	0.31	0.05	0.05	0.81
Bonferroni post hoc P - value		-	-	-	-	-
CC	Correct	113.6 ± 16.7	104.4 ±6.7	96.9 ± 24.0	112.0 ± 7.0	120.7 ± 5.3
CC	Narrow	109.9 ± 28.4	95.8 ± 23.8	98.6 ± 26.6	99.0 ± 26.8	107.7 ± 30.9
CC	Wide	106.5 ± 37.4	93.5 ± 34.5	94.7 ± 39.1	95.0 ± 38.0	104.5 ± 42.2
P – value		0.81	0.42	0.92	0.20	0.29
Bonferroni post hoc P - value		-	-	-	-	-

Table S4. - Displaying IMU kinematic data whilst cantering for each saddle condition, correct, narrow and wide. Significance level set at $p \leq 0.05$ with a Bonferroni post hoc correction to determine pairwise significance between conditions with only significant post hoc results being presented (Bonferroni adjusted alpha 5%). (FE=flexion-extension, AR = Axial Rotation, LB = Lateral Bending).

**Thoracolumbar Spine Rotations**	**Saddle Condition**	**T5 Mean ± SD (°)**	**T13 Mean ± SD (°)**	**T18 Mean ± SD (°)**	**L3 Mean ± SD (°)**	**TS Mean ± SD (°)**
FE	Correct	15.6 ± 1.9	9.2 ± 1.73	14.8 ± 6.3	12.2 ± 1.5	14.3 ± 1.8
FE	Narrow	14.8 ± 4.3	9.6 ± 2.31	11.7 ± 3.2	12.1 ± 2.5	13.4 ± 2.8
FE	Wide	13.4 ± 4.0	9.0 ± 6.72	11.1 ± 3.6	12.5 ± 3.8	13.2 ± 3.6

P - value		0.30	0.61	0.03	0.90	0.53
Bonferroni post hoc P - value		-	-	correct – wide, 0.04	-	
AR	Correct	13.4 ± 4.4	12.1 ±2.9	20.5 ± 5.2	16.5 ± 2.8	13.3 ± 3.1
AR	Narrow	13.8 ± 2.9	10.9 ± 3.4	20.7 ± 6.0	14.1 ± 2.9	13.2 ± 3.3
AR	Wide	16.2 ± 3.7	8.9 ± 2.6	20.2 ± 6.0	14.0 ± 3.6	14.1 ± 2.5
P - value		0.02	0.01	-	0.007	0.11
Bonferroni post hoc P - value		correct - wide, 0.03	correct – wide, 0.04		correct – wide, 0.03	
LB	Correct	8.9 ± 3.8	6.2 ±1.5	10.9 ± 4.9	7.3 ± 3.0	6.6 ± 2.2
LB	Narrow	7.7 ± 1.2	6.1 ± 1.6	7.9 ± 2.3	6.1 ± 1.6	6.4 ± 1.5
LB	Wide	9.0 ± 2.4	5.7 ± 1.4	9.0 ± 2.3	6.1 ± 2.4	6.0 ± 1.6
P - value		0.54	0.72	0.03	0.32	0.56
Bonferroni post hoc P - value		-	-	-	-	-
